# Perceived clinical utility of a test for predicting inadequate response to TNF inhibitor therapies in rheumatoid arthritis: results from a decision impact study

**DOI:** 10.1007/s00296-020-04746-7

**Published:** 2020-11-30

**Authors:** Dimitrios A. Pappas, Christine Brittle, James E. Mossell, Johanna B. Withers, Jeraldine Lim-Harashima, Joel M. Kremer

**Affiliations:** 1 Corrona LLC, Waltham, MA USA; 2 Corrona Research Foundation, Waltham, MA USA; 3 Tift Regional Medical Center, Tifton, GA USA; 4 HealthiVibe, a Division of Corrona LLC, Arlington, VA USA; 5 Scipher Medicine Corporation, Waltham, MA USA

**Keywords:** Arthritis, Rheumatoid, Surveys and questionnaires, Decision-making, Tumor necrosis factor inhibitors, Precision medicine, Predictive value of tests, Therapeutics

## Abstract

Tumor necrosis factor inhibitor (TNFi) therapies are often the first biologic therapy used to treat rheumatoid arthritis (RA) patients. However, a substantial fraction of patients do not respond adequately to TNFi therapies. A test with the ability to predict response would inform therapeutic decision-making and improve clinical and financial outcomes. A 32-question decision-impact survey was conducted with 248 rheumatologists to gauge the perceived clinical utility of a novel test that predicts inadequate response to TNFi therapies in RA patients. Participants were informed about the predictive characteristics of the test and asked to indicate prescribing decisions based on four result scenarios. Overall, rheumatologists had a favorable view of the test: 80.2% agreed that it would improve medical decision-making, 92.3% said it would increase their confidence when making prescribing decisions, and 81.5% said it would be useful when considering TNFi therapies. Rheumatologists would be more likely to prescribe a TNFi therapy when the test reported that no signal of non-response was detected (79.8%) and less likely to prescribe a TNFi therapy when a signal of non-response was detected (11.3%–25.4%). Rheumatologists (84.7%) agreed that payers should provide coverage for such a test. This study shows that rheumatologists support the clinical need for a test to predict inadequate response to TNFi therapies. Test results were perceived to lead to changes in prescribing behaviors as results instill confidence in the ordering rheumatologist.

**Electronic supplementary material:**

The online version of this article (10.1007/s00296-020-04746-7) contains supplementary material, which is available to authorized users.

## Introduction

Rheumatoid arthritis (RA) is a chronic autoimmune disease that affects about 1.3 million U.S. adults [1]. When RA is not adequately controlled, joint damage and chronic inflammation can lead to permanent disability and poor health outcomes, including shortened life expectancy. Often, multiple treatment cycles may be needed to find the appropriate therapeutic for an individual patient before adequate disease control is achieved. Such a “trial-and-error” approach increases overall costs while decreasing patient satisfaction [2].

The American College of Rheumatology (ACR) guidelines support the use of any targeted therapy, regardless of MOA, for the treatment of RA following inadequate response to a csDMARD [3]. Tumor necrosis factor inhibitor (TNFi) therapies are usually the first biologic tried after the failure of csDMARDs [4]. This prescribing behavior is reinforced by the current medical policies of insurers, which mostly cover TNFi therapies as the only first-tier treatment following csDMARD failure [5–8]. Despite being widely used, TNFi therapies are not always effective. Clinical studies show more than half of patients fail to achieve an ACR50 response (indicating a 50% improvement in disease activity) or meaningful clinical change [9, 10]. Precision medicine can alter this treatment paradigm to better meet the needs of individual patients, as has happened in other medicine fields, such as oncology [11–13].

In the absence of predictive markers to inform individual treatment decisions, rheumatologists have largely made drug selections based on insurance coverage or other drivers, such as habit and their familiarity with specific therapeutics. Precision medicine has the promise to change RA treatment using biomarkers to target drugs to patients based on their likely effectiveness [2, 14]. For example, knowing that the likelihood of non-response to a TNFi is high may lead to prescription of an alternative biologic.

Currently, when an initial TNFi therapy fails, patients may be placed on alternate TNFi therapy, a process known as TNFi cycling [15]. For a significant proportion of these patients, adequate disease control will not be achieved, thus leading to prolonged patient symptoms, loss of function, and frustration [15–17]. The European League Against Rheumatism (EULAR) acknowledges that a weakness in the current RA treatment paradigm is the absence of a method to stratify patients to the most appropriate treatment [16]. Thus, there is a need for a predictive test to identify which patients may be unlikely to have an adequate response to specific biologics.

Such a test (PrismRA) was made clinically available after this survey concluded and predicts inadequate response to TNFi therapies for RA patients with a positive predictive value of 89.7%, a specificity of 86.8%, and a sensitivity of 50%.

This decision-impact study was conducted to evaluate rheumatologists’ insights on the value and perceived clinical utility of a precision medicine test that predicts inadequate response to TNFi therapies for RA patients. Rheumatologists were asked to share their opinions about the inability to predict inadequate response to TNFi therapies, evaluate the characteristics of a test that would alleviate this issue, and investigate the possible clinical utility of such a test. A decision-impact study is important, because it is not known how rheumatologists would implement such a test. A large observational study of rheumatologists found that “physician preference was a significant determinant” of use of specific biologics, “independent of demographic and other clinical factors” [18]. Predictive tests of response may alter prescription patterns, and our study aimed to shed light on how such tests will be perceived by rheumatologists.

## Methods

Data were collected via a cross-sectional survey of U.S. rheumatologists. A 32-item decision-impact survey was designed by HealthiVibe, a division of Corrona, LLC, a research and consulting company. The survey was conducted from May 28 to June 11, 2020 using the online survey platform SurveyGizmo. On average, the survey took 12 min to complete. The survey instrument was cognitively pretested with seven practicing rheumatologists using a think-aloud technique prior to being finalized, to ensure content and construct validity. The complete survey instrument is available electronically as supplemental information to this article (Supplement 1). The study was reviewed by the Sterling Institutional Review Board, and a letter of exemption as non-human subjects research was received. All respondents were asked to review an informed consent statement prior to participating. Respondents were advised that participation was voluntary and that they could withdraw at any time.

### Participants

Rheumatologists were recruited from three panels (M3 Global Research, Exact Data, and the Corrona RA registry) and offered a small gratuity ($20–$35) for completing the survey. Respondents were told that the purpose of the survey was to seek “feedback from physicians about their treatment of rheumatoid arthritis patients” and were instructed to answer reflecting practice patterns prior to COVID-19 so that responses reflected normal practice. Study qualification requirements were set to reflect rheumatologists who may utilize a predictive test for TNFi therapy response. Criteria included: a primary medical specialty of rheumatology; primarily treat adult patients; evaluate at least 15 RA patients per month; at least 10% of RA patients are biologic-naïve; and initiate a new prescription for a biologic or JAK inhibitor at least once every 3 months.

### Survey methodology

Questions were organized into three main sections. The first collected demographic information and addressed rheumatologists’ attitudes and prescribing patterns regarding treatment of RA patients with biologics and JAK inhibitors. This included demographic and practice setting-related questions. It also included questions about response to TNFi therapies. Rheumatologists were asked to rate their level of concern about various issues related to inadequate response.

**Fig. 1:**
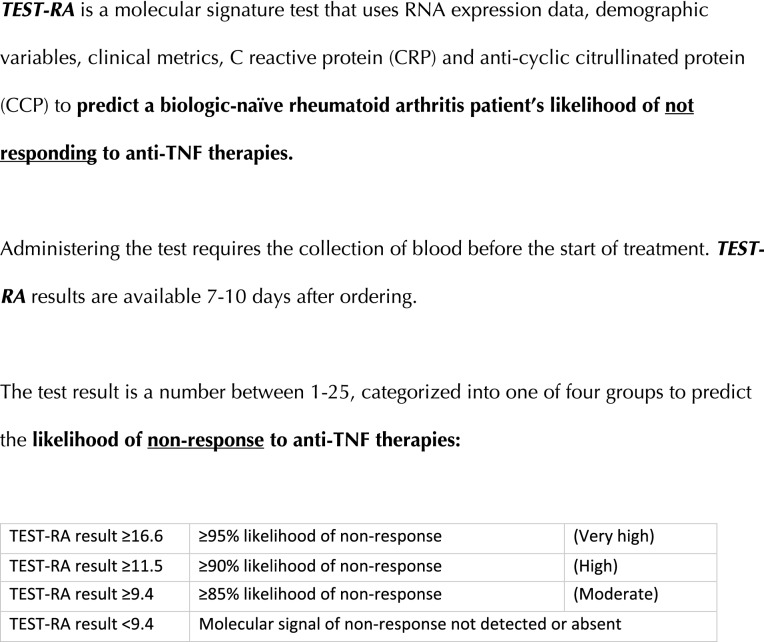
Description of a molecular signature test that predicts a patient’s likelihood of inadequate response to TNFi therapies that was shared with survey respondents

The second section introduced rheumatologists to a commercial test that predicts inadequate response to TNFi therapies. Rheumatologists were provided with a brief test description, but were not provided with detailed test specifications. The test name and manufacturer information were also not provided to reduce commercial bias and conflicts of interest. The test was referred to as *TEST-RA *throughout the survey. *TEST-RA* was described as “a molecular signature test that uses RNA expression data, demographic variables, clinical metrics, C-reactive protein (CRP) and anti-cyclic citrullinated protein (CCP) to predict a biologic-naïve rheumatoid arthritis patient’s likelihood of not responding to anti-TNF therapies” (Fig. 1). After being introduced to *TEST-RA*, survey respondents rated their level of agreement with 12 statements about the test.

**Fig. 2:**
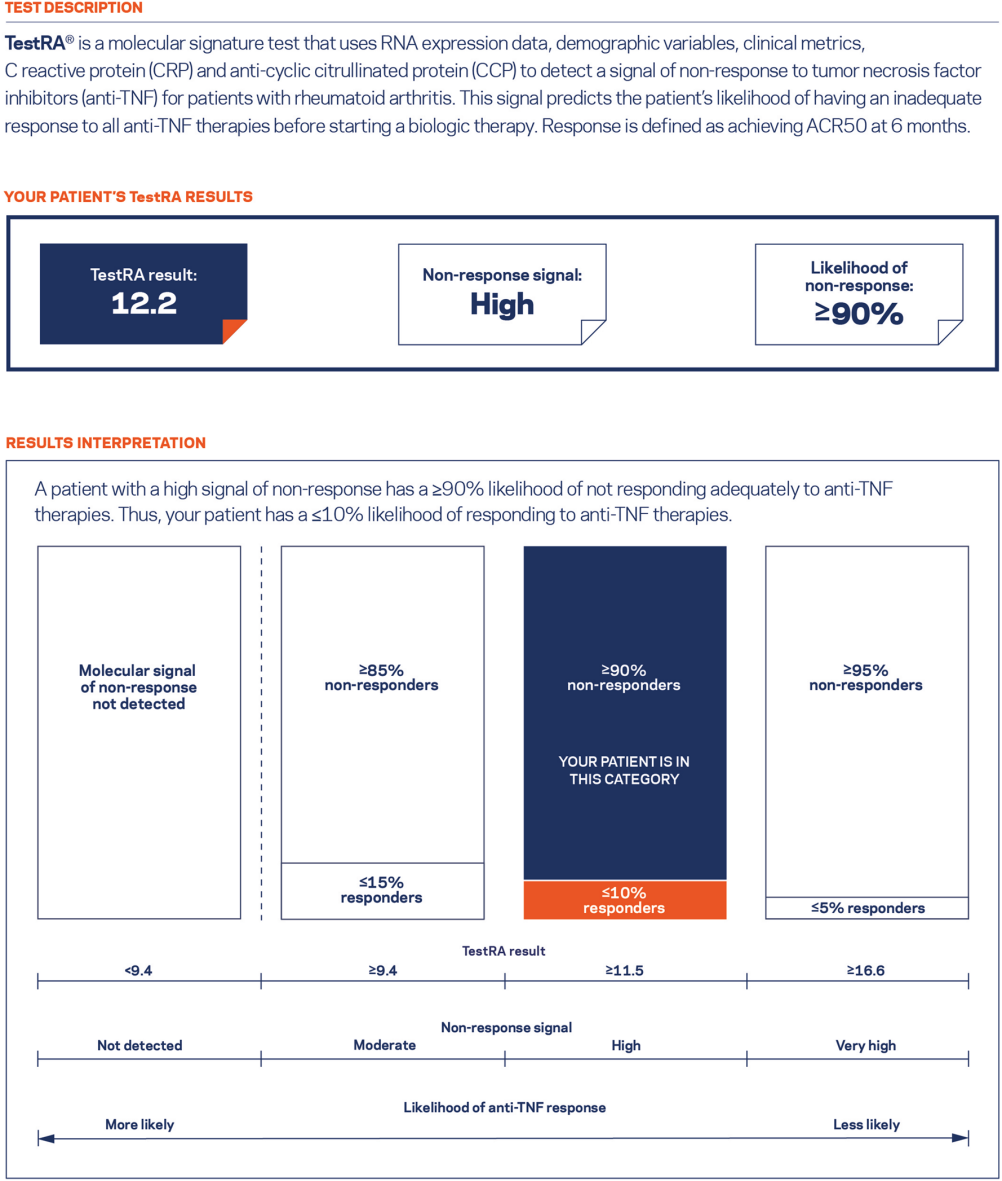
Sample test result shown to respondents. The version describing the high signal of non-response to TNFi therapies is depicted as a representative example. Three additional sample reports were shown to respondents with the patient *TEST-RA* results changed to those corresponding to no signal, moderate signal, or very high signal of non-response

The third section evaluated the value and impact of *TEST-RA* including the likely decision impact of such a test. Based on reactions to four different scenarios depicting sample test results (Fig. 2), rheumatologists were asked to indicate their prescribing behaviors. The scenarios were shown to respondents in random order to reduce bias.

Descriptive statistics were used to characterize trends in the data. Key survey variables were evaluated in a cross-tabulation analysis against demographic variables including years in practice, number of RA patients seen per month, gender, race, practice characteristics, and practice setting. Chi-square analyses were used to identify statistically significant differences across respondent groups. No weighting of survey data was performed.

## Results

### Participant demographics

A total of 467 respondents began the survey; 205 were excluded due to ineligibility, resulting in an eligibility rate of 56.1% (262). Among the 43.9% who were ineligible: 70.8% had a specialty other than rheumatology; 22.8% saw less than 10% of patients who are biologic-naïve; 3.9% primarily saw pediatric patients; and 2.4% screened out for other reasons. Among eligible respondents, almost all (248/262; 94.7%) completed the survey.

As shown in Table 1, participants were primarily male (66.5%) and white (58.9%). Most (59.3%) had been in practice 11 or more years, and more than half (52.8%) saw 81 or more adult RA patients per month. About a third (32.7%) practiced in an academic setting. These results are similar to the demographics reported in the 2015 ACR Workforce Study [19], which found that 59.2% of rheumatologists are male, and 20% practice in an academic setting.

**Table 1:** Demographics and practice characteristics of survey respondents

	%	*n*
Gender		
Male	66.5	165
Race or ethnicity		
White	58.9	146
Black	2.4	6
Hispanic or Latino	2.8	7
Asian	31.1	77
Other	6.1	15
Years in practice (since fellowship)		
Less than 2 years	4.8	12
3 to 5 years	10.5	26
6 to 10 years	25.4	63
11 to 20 years	33.9	84
More than 20 years	25.4	63
Adult RA patients per month		
15 to 40	12.9	32
41 to 80	34.3	85
81 or more	52.8	131
Practice setting		
Academic	32.7	81
Non-academic	67.3	167
Practice type		
Solo	13.7	34
Single specialty	35.1	87
Multispecialty	51.2	127
Practice affiliations		
Connected to hospital/hosp. system	48.0	119
Part of an IDN	12.1	30
Part of an ACO	13.7	34
Geographic location		
Rural	5.2	13
Suburban	44.4	110
Urban	50.4	125
U.S. region		
Midwest	18.2	45
Northeast	29.4	73
Southeast	23.8	59
Southwest	10.5	26
West	18.2	45
Self-identified early adopter of medical advances		
Yes	77.8	193

### Concerns about inadequate response to TNFi therapies

Participants were asked a series of questions to gauge their attitude about inadequate response to TNFi therapies in RA. Published studies report a 32–38% rate of low disease activity (LDA) or remission in response to TNFi therapies [20–22]; in this survey, 79.4% of rheumatologists believed that more than 30% of their RA patients prescribed a TNFi therapy reached LDA or remission (Table 2). For patients who do not adequately respond to TNFi therapies, the majority of rheumatologists expressed concern about the increased time for those patients to achieve remission or low disease activity (73.0%), patients paying for drugs that are not helping them reach treatment targets (71.0%), the difficulty getting alternatives to TNFi therapies approved by payers (65.7%), and reduced patient satisfaction (64.1%) (Table 2). Consistent with these concerns, approximately two-thirds (67.7%) of rheumatologists were concerned about the difficulty of predicting which patients will be non-responders to TNFi therapies, and 98.8% expressed interest in a test to predict inadequate response to TNFi therapies in RA patients (Table 2).

**Table 2:** Rheumatologists’ current approaches to and attitudes about inadequate response to TNFi therapies

	%	*n*
Percentage of patients believed by rheumatologist to respond adequately to an initial TNFi therapy		
Less than 20%	0.8	2
21–39%	19.8	49
40–59%	51.2	127
60% or more	28.2	70
The percent of rheumatologists who are concerned or very concerned about five issues related to inadequate response to TNFi therapies		
Non-response increasing time to low disease activity state	73.0	181
Patients paying for drugs not getting them to targets	71.0	176
Difficulty predicting which patients will be non-responders	67.7	168
Difficulty getting other drugs approved by payers/plans	65.7	163
Non-responders having reduced patient satisfaction	64.1	159
Interest in a test to predict inadequate response to TNFi therapies		
Not at all interested	1.2	3
Slightly interested	2.0	5
Moderately interested	11.3	28
Very interested	41.5	103
Extremely interested	44.0	109

### Reactions to TEST-RA

**Fig. 3:**
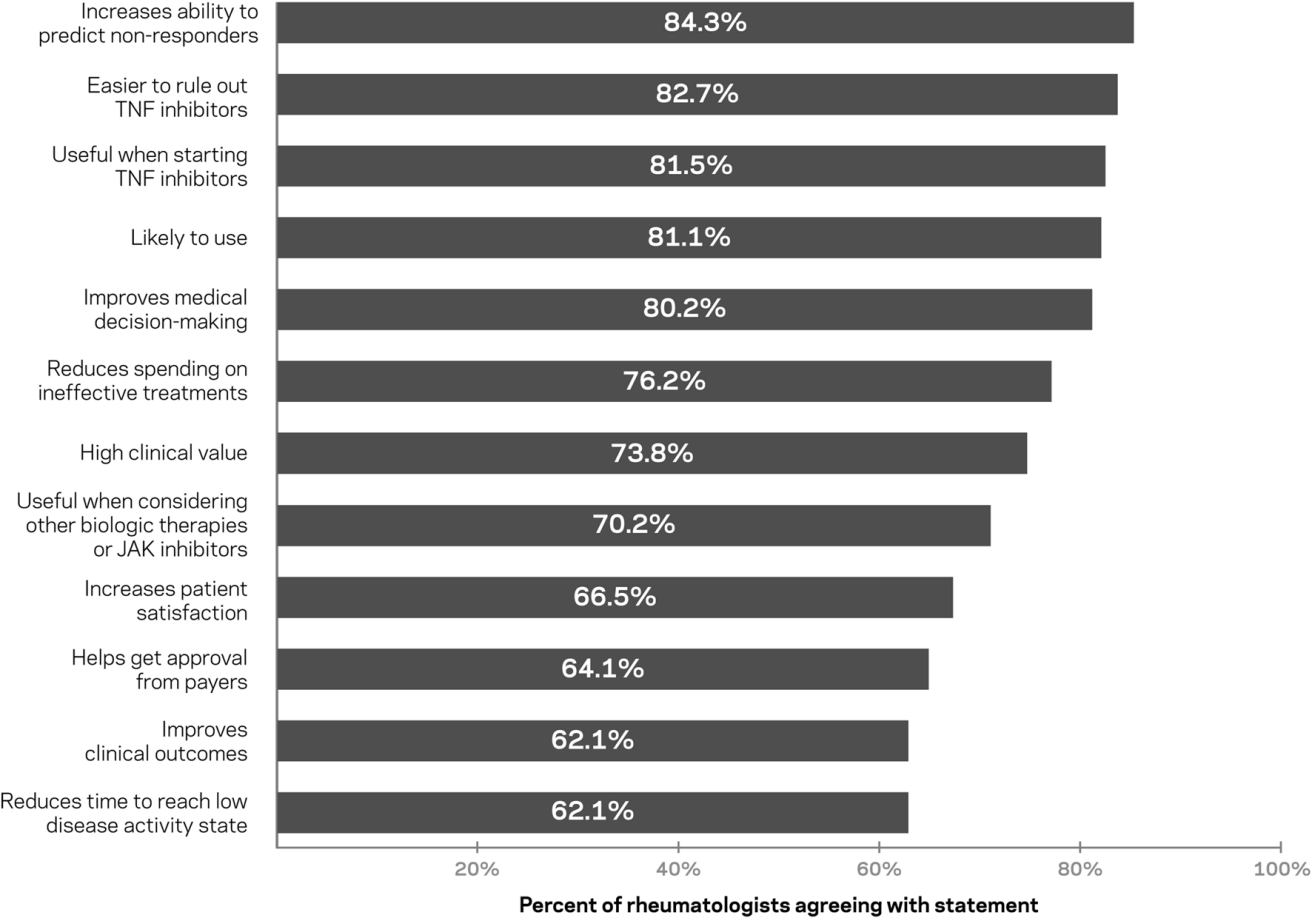
Percentage of rheumatologists agreeing with statements about a test to predict inadequate response to TNFi therapies

**Fig. 4:**
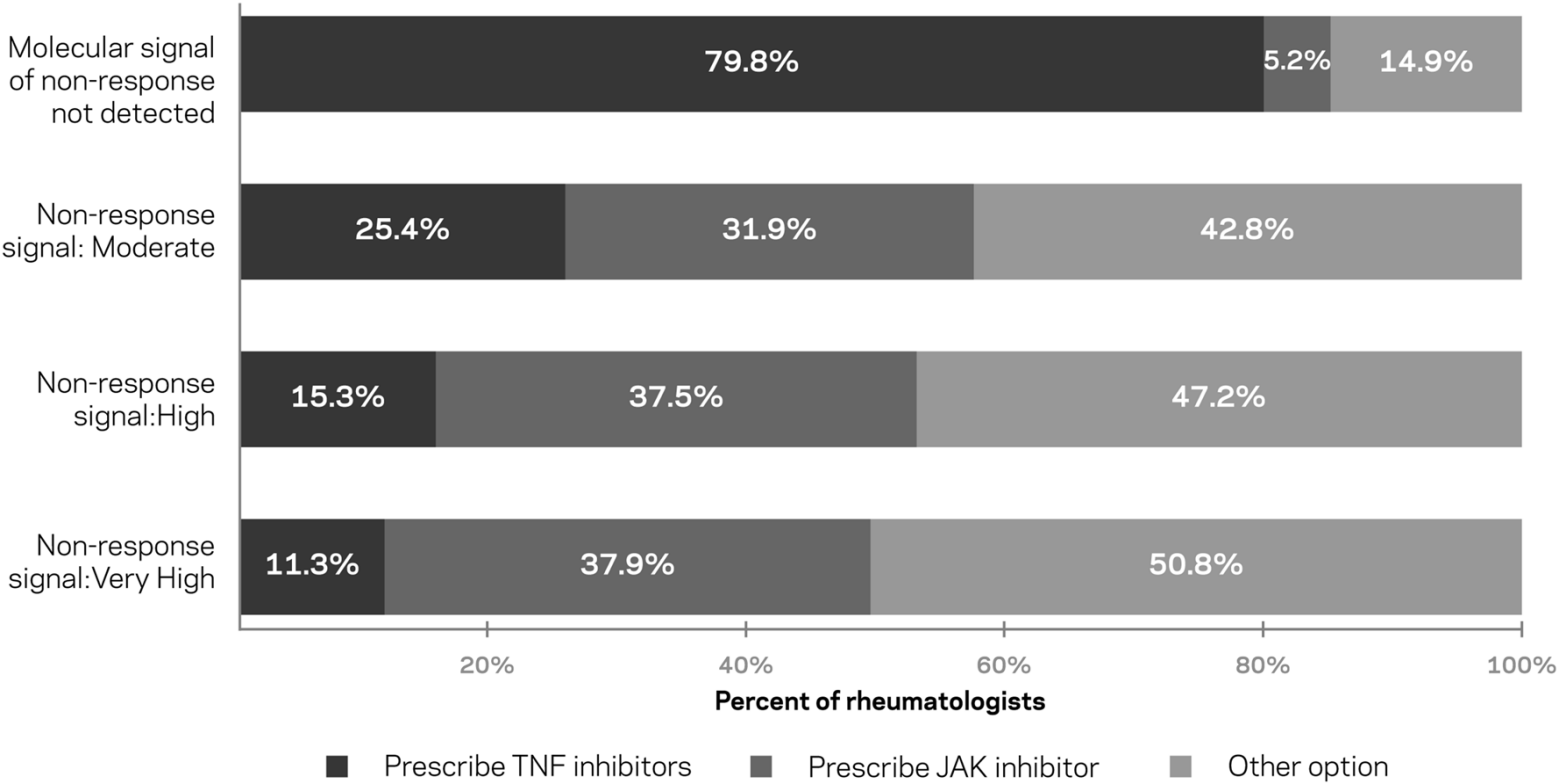
Prescribing behavior of rheumatologists based on four sample test results that report a patient’s likelihood of having an inadequate response to TNFi therapies (no signal, moderate signal, high signal, and very high signal of non-response). Other options included IL-6 receptor antagonist, T cell co-stimulation inhibitor, B cell inhibitor, IL-1 receptor antagonist, and maximizing patient’s current medication

Rheumatologists were provided information on *TEST-RA *(Fig. 1) and asked to provide their reactions on issues, such as ineffective medication spend, patient satisfaction, insurance coverage, and improved outcomes, such as low disease activity (Fig. 3). The majority agreed with statements about the usefulness of *TEST-RA.* Over 80% agreed that it increases their ability to predict non-response to TNFi therapies (84.3%) and makes it easier to rule out TNFi therapies (82.7%), and 70.2% agreed on its usefulness when considering other biologic therapies or JAK inhibitors. Additionally, the majority of the respondents agreed with clinical utility statements on *TEST-RA.* About 80% agreed that it will improve medical decision-making, 76.2% agreed it will reduce spending on ineffective treatments, and 81.5% agreed results will be useful when considering starting a patient on TNFi therapy. Finally, the majority of respondents agreed with statements about the value of *TEST-RA.* About 81% agreed that they would be likely to use the test, while almost 74% agreed the test has a high clinical value. Cross-tabulation analyses showed that agreement with these statements was largely consistent across demographic groups (Fig. 4).

Most rheumatologists surveyed (92.3%) agreed that *TEST-RA* would increase their confidence in making prescribing decisions, and nearly 85% believed that payers should provide full coverage for *TEST-RA.* Moreover, almost all (98.4%) said the test was helpful in some way when deciding whether to start a patient on TNFi therapy.

### TEST-RA results impact prescribing decisions

Respondents were shown four different sample *TEST-RA* results in random order (see example in Fig. 2), which corresponded to the four possible tiers of predicted inadequate response to TNFi therapies per *TEST-RA*: no signal; moderate signal (≥ 85% likelihood of non-response); high signal (≥ 90% likelihood of non-response); and very high signal (≥ 95% likelihood of non-response). Rheumatologists were asked to indicate what therapies they would prescribe based on each test result. Options included: TNFi; IL-6 receptor antagonist; JAK inhibitor; T cell co-stimulation inhibitor; B cell inhibitor; and IL-1 receptor antagonist.

In response to the *TEST-RA* sample reports, rheumatologists reported the following prescribing decisions: When *TEST-RA* reported that a molecular signal of non-response to TNFi therapies was not detected, 79.8% of rheumatologists would prescribe a TNFi therapy. In contrast, when a molecular signal of non-response to TNFi therapies was present, rheumatologists were less likely to prescribe a TNFi therapy, and the likelihood decreased as the strength of the reported non-response signal increased (moderate: 25.4%; high: 15.3%; very high: 11.3%).

Most rheumatologists accurately interpreted the *TEST-RA* results according to the intended meaning. However, a small number (6.7%) provided answers that were consistent with an error in comprehension. For example, they indicated they would not prescribe a TNFi therapy if the test showed a “moderate” signal of non-response, but they would prescribe a TNFi therapy if the signal was “high” and/or “very high.” When these respondents were removed from the analysis, about three-quarters (73.6%; 170/231) of the remaining respondents indicated that they would not prescribe a TNFi therapy if any signal of non-response was detected. An additional 13.9% would not prescribe a TNFi therapy if the signal was “high” or “very high.” Thus, 87.4% (202/231) would not prescribe a TNFi therapy if the signal of non-response was “high” or “very high.”

## Discussion

This decision-impact study evaluated rheumatologists’ perceptions and interpretations of a novel molecular signature test that identifies predicted inadequate responders to TNFi therapies. This test identifies with close to 90% accuracy half of RA patients who will not have an ACR50 response to TNFi therapies by 6 months [23].

In this study, rheumatologists expressed their concerns regarding the inability to predict “non-responder” patients and the clinical consequences of inadequate response to TNFi therapies. Almost all rheumatologists (98.8%) expressed interest in a test that predicts which patients will not have an adequate response to TNFi therapies and, when presented with sample test reports, indicated that the results would adjust their treatment decisions and medical management of RA patients. Rheumatologists reported that they would be less likely to prescribe TNFi therapies as the strength of the molecular signal of inadequate response increased.

A substantial clinical and economic burden is associated with the treatment of RA. One of the many challenges facing patients with RA and their physicians is deciding when to initiate targeted therapy and which medication class to select. The prescribing pattern of selecting TNFi therapy first after the failure of csDMARDs is a combination of formulary restrictions, rebate-driven pricing strategies, and habit. In this study, 71.0% of rheumatologists were concerned that inadequate response to TNFi therapy means that patients are paying for drugs that do not get them to treatment targets.

Current medical policies and prescribing patterns may result in patients cycling through multiple rounds of TNFi medications before they can select a drug with a different mechanism of action. Cycling increases healthcare and out-of-pocket costs when patients do not respond [24, 25]. Furthermore, continuing to use a medication class that is not effective results in higher disease activity and reduces the patient’s quality of life [26]. After patients fail their first TNFi therapy, they are 27% more likely to inadequately respond to their second medication and three times more likely to discontinue therapy [27]. They incur more joint surgeries [28] and have a higher likelihood of irreversible joint damage and chronic pain [29]. Thus, it is critically important to correctly and quickly identify effective treatments for RA patients.

The results of this study reflected rheumatologists’ clear interest in the value and clinical utility of a test that predicts inadequate response to TNFi therapies in RA. As has been done for other biomarker tests, this decision-impact survey demonstrated the clinical value of a molecular signature test that predicts inadequate response to TNFi therapies [30, 31]. Based on an initial description and sample results, rheumatologists indicated the test results would alter their medical management of RA patients. Nearly 85% of rheumatologists believed that insurance companies should provide full coverage for a test that predicts inadequate response to TNFi therapies in RA. This finding may express the need for new technology being accessible to healthcare providers so they can play an active role in reducing wasteful spending on ineffective treatments.

This decision-impact study has limitations to consider. Rheumatologists were provided a short description of the molecular signature test (Fig. 1) and did not have a chance to review clinical evidence or data supporting its development and validation. This may have contributed to some of the misunderstandings observed in this survey among rheumatologists who appear to have contradicted themselves on different data points. In addition, rheumatologists were not provided information on the cost of the test or its likelihood to be covered by insurance. Cost and coverage are likely to impact real-world use or adoption of such a test. As it is customary, rheumatologists were also offered a small stipend for participating in the decision-impact survey. This could also have influenced responses. While rheumatologists expressed interest in a test like *TEST-RA*, this cannot, of course, be interpreted as a direct endorsement of *TEST-RA*.

In conclusion, this study showed the need for predictive response tests in rheumatology and suggests that a test that predicts inadequate response to TNFi therapies has perceived clinical utility while providing meaningful new information for patient stratification in RA. Professional societies have long identified the need to tailor therapy approaches to individual patients [16] and to adopt a personalized, precision medicine approach in rheumatology. Pioneer specialties in precision medicine—oncology and hematology—have improved patient outcomes by adjusting therapy choice based on patient and tumor characteristics. The introduction of precision medicine would be welcomed by the rheumatology community, test results would lead to treatment changes, and patient care would improve by avoiding a medication class that would not result in meaningful change for those patients predicted to be inadequate responders.

## Electronic supplementary material

Below is the link to the electronic supplementary material.

Electronic supplementary material 1 (PDF 292 kb)
