# Supplementary material for: Perceived clinical utility of a test for predicting inadequate response to TNF inhibitor therapies in rheumatoid arthritis: results from a decision impact study
Source: Rheumatol Int. 2020 Nov 30;41(3):585–93. doi: 10.1007/s00296-020-04746-7 (PMC7703510; doi:10.1007/s00296-020-04746-7)
Supplement: Supplementary file 1 — Electronic supplementary material 1 (PDF 292 kb) [file 296_2020_4746_MOESM1_ESM.pdf]

Article title: Perceived clinical utility of a molecular signature test for predicting inadequate response to TNF inhibitor therapies in rheumatoid arthritis

Journal name: Rheumatology International

Author names:
Dimitrios A. Pappas, M.D., MPH
Christine Brittle, Ph.D.
James E Mossell III, D.O., F.A.C.R.
Johanna Withers, Ph.D.
Jeraldine Lim-Harashima, MS CGC
Joel M Kremer, MD

Affiliation and e-mail address of the corresponding author:
Corrona LLC, Waltham, MA, USA
dpappas@corrona.org

Supplemental Material 1 Survey questionnaire completed by rheumatologists

Rheumatologist Perspectives Survey

Thank you for your interest in this survey. We are seeking feedback from physicians about their treatment of rheumatoid arthritis patients.

By entering the survey link you understand and agree to the following:

- I understand that the aim of this research is to gain my views for market research purposes in the development of marketing campaigns, educational materials and the development of new commercial products AND IS NOT INTENDED AS A PROMOTIONAL EXERCISE.
- I agree that anything I see or read during this research should be treated as confidential. Any information presented during the course of this research is done solely to explore reactions to such information and should be assumed to represent hypotheses about what can be said about a product or disease area. It should not be used to influence decisions outside the research setting.
- I understand that I can withdraw at any time.
- I understand that any information I disclose will be treated in the strictest confidence and that the results of the research will be aggregated to provide an overall picture of attitudes. My feedback will remain confidential. My personal information will not be passed to any other organization without my permission.

() I accept these conditions for taking the survey.

() I do not accept these conditions for taking the survey. **[TERMINATE]**

Thank you. We will begin with a few questions about you and your practice. **Please answer the questions reflective of your typical practice patterns prior to COVID-19.**

1. What is your primary medical specialty?
   () Rheumatology
   () Family Practice/Primary Care **[TERMINATE]**
   () Internal Medicine **[TERMINATE]**
   () Other **[TERMINATE]**

2. How many years have you been in clinical rheumatology practice (time since completing fellowship)?
   () Less than 2 years
   () 3 to 5 years
   () 6 to 10 years
   () 11 to 20 years
   () More than 20 years

3. Which region of the country do you practice in?
   () Midwest: IA, IL, IN, KS, MI, MN, MO, ND, NE, OH, SD, WI
   () Northeast: CT, DC, DE, MA, MD, ME, NH, NJ, NY, PA, RI, VT
   () Southeast: AL, AR, FL, GA, KY, LA, MS, NC, SC, TN, VA, WV
   () Southwest: AZ, NM, OK, TX
   () West: AK, CA, CO, HI, ID, MT, NV, OR, UT, WA, WY

4. What percentage of your patients are pediatric (aged 17 and under)?
   () None (0%)
   () 1-10%
   () 11-50%
   () 51% or higher **[TERMINATE]**

5. How many adult **rheumatoid arthritis** patients do you personally see per month?
   () 14 or fewer **[TERMINATE]**
   () 15 to 40
   () 41 to 80
   () 81 or more

6. Among all your rheumatoid arthritis patients, about what percentage are **biologic-naïve**?
   () 0-9% **[TERMINATE]**
   () 10-19%
   () 20-29%
   () 30-39%

() 40-49%
() 50% or higher

7. How often do you typically initiate a new prescription for a biologic or JAK inhibitor for a rheumatoid arthritis patient?
   () At least once a week
   () At least once a month, but not every week
   () At least once every three months, but not every month
   () Rarely, less often than every three months **[TERMINATE]**

Thank you. The following questions are about your treatment approach for rheumatoid arthritis patients. **Please check yes to verify that your responses will reflect your treatment strategies prior to COVID-19.**

() Yes, my responses will reflect my treatment strategies prior to COVID-19 **[REQUIRE YES TO CONTINUE SURVEY]**

8. Do you consider yourself an early adopter of medical advances, meaning you are likely to adopt new diagnostic tests, clinical guidelines, and medications before most of your peers?
   () No
   () Yes

9. Do you believe available clinical and laboratory information are sufficient to **predict** whether rheumatoid arthritis patients will achieve adequate response (defined as low disease activity or remission) to an anti-TNF therapy?
   () No
   () Yes

10. What percentage of your patients **respond adequately** (defined as low disease activity or remission) to an initial anti-TNF therapy?
   () Less than 20%
   () 21-39%
   () 40-59%
   () 60% or more

11. For a patient who fails to respond adequately to initial anti-TNF therapy, what would you be most likely to do next?
   () Switch to an alternate anti-TNF therapy (e.g., adalimumab, certolizumab pegol, etanercept, golimumab, or infliximab)
   () Switch to an IL-6 receptor antagonist (e.g., sarilumab or tocilizumab)
   () Switch to a JAK inhibitor (e.g., baricitinib, tofacitinib, or upadacitinib)
   () Switch to a T cell co-stimulation inhibitor (e.g., abatacept)

() Switch to a B cell inhibitor (e.g., rituximab)
() Switch to an IL-1 receptor antagonist (e.g., anakinra)
() Maintain original anti-TNF, but add additional medication (e.g., conventional synthetic DMARDs or steroids)
() Maintain original anti-TNF, but modify dose
() Something else

12. How long do you typically wait to determine if a patient has responded adequately to an initial anti-TNF therapy (i.e., how long before deciding whether to make a change)?
() Less than 8 weeks
() 8 to 12 weeks
() 13 to 16 weeks
() 4 to 6 months
() 7 months or more

13. Thinking about **anti-TNF therapy non-response**, how concerned are you about each of the following? **[ROTATE]**

	Not at all concerned	Somewhat concerned	Concerned	Very concerned
Difficulty predicting which patients will be non-responders				
Non-response increasing the time to eventually reach remission or low disease activity state				
Non-responders having reduced patient satisfaction				
Difficulty getting a drug other than an anti-TNF therapy approved by payers/plans				
Patients paying for drugs that are not getting them to treatment targets				

14. How interested would you be in a tool or test to predict anti-TNF therapy non-responders?
() Not at all interested
() Slightly interested
() Moderately interested
() Very interested
() Extremely interested

15. Which of the following best describes how you feel about a molecular signature (defined as a patient's specific genes, proteins, or genetic variables that can be used as biomarkers) and its relationship to drug therapy?
() A patient's molecular signature has **high value** in predicting their response to drug therapy
() A patient's molecular signature has **moderate value** in predicting their response to drug therapy
() A patient's molecular signature has **limited value** in predicting their response to drug therapy
() I am not familiar enough with molecular signature to have an opinion

Please read the following description of a molecular signature test being developed for rheumatoid arthritis patients.

> ***TEST-RA*** *is a molecular signature test that uses RNA expression data, demographic variables, clinical metrics, C reactive protein (CRP) and anti-cyclic citrullinated protein (CCP) to* ***predict a biologic-naïve rheumatoid arthritis patient's likelihood of <u>not responding</u> to anti-TNF therapies.***
>
> *Administering the test requires the collection of blood before the start of treatment.* ***TEST-RA*** *results are available 7-10 days after ordering.*
>
> *The test result is a number between 1-25, categorized into one of four groups to predict the* ***likelihood of <u>non-response</u> to anti-TNF therapies:***

TEST-RA result ≥16.6	*≥95% likelihood of non-response*	*(Very high)*
TEST-RA result ≥11.5	*≥90% likelihood of non-response*	*(High)*
TEST-RA result ≥9.4	*≥85% likelihood of non-response*	*(Moderate)*
TEST-RA result <9.4	*Molecular signal of non-response not detected or absent*	

Based on this description, please answer the following questions.

16. How much do you agree or disagree with each of the following statements? **[ROTATE]**

	Completely disagree	Somewhat disagree	Neither agree nor disagree	Somewhat agree	Completely agree
TEST-RA has a high clinical value					
TEST-RA improves clinical outcomes					
I am likely to use ***TEST-RA***					
TEST-RA improves medical decision-making					

TEST-RA helps provide the information I need to get approval for drugs from payers					
TEST-RA results are useful when considering starting an anti-TNF therapy					
TEST-RA results are useful when considering starting other biologic therapies (not anti-TNFs) or a JAK inhibitor					

17. What are your initial reactions to ***TEST-RA***? Please describe below.

    _____

    _____

    _____

18. For what percentage of biologic-naïve patients would you use ***TEST-RA*** before prescribing an anti-TNF therapy?
    () None (0%)
    () 1-19%
    () 20-39%
    () 40-59%
    () 60-79%
    () 80-99%
    () All (100%)

19. **[FOR ALL ANSWERS ABOVE EXCEPT 100%]** You indicated that you would not use ***TEST-RA*** on every patient. Why would you not use ***TEST-RA*** on every patient prior to prescribing an anti-TNF therapy? Please describe below.

    _____

    _____

    _____

Next, we will show you a series of patient results for ***TEST-RA***.

Please review this result, and then indicate your response.
[SHOW TEXT ABOVE FOLLOWED BY IMAGE OF TEST RESULT. ROTATE.]

20. Assume you had gotten these ***TEST-RA*** results for a patient you were considering starting on an anti-TNF therapy. Based on these results, what would you be most likely to do next?
    () Start patient on an anti-TNF

() Start patient on an IL-6 receptor antagonist (e.g., sarilumab or tocilizumab)
() Start patient on a JAK inhibitor (e.g., baricitinib, tofacitinib, or upadacitinib)
() Start patient on a T cell co-stimulation inhibitor (e.g., abatacept)
() Start patient on a B cell inhibitor (e.g., rituximab)
() Start patient on an IL-1 receptor antagonist (e.g., anakinra)
() Maximize patient's current medication(s)
() Something else

[REPEAT QUESTION FOR EACH IMAGE]

Thanks. Now we will ask about ***TEST-RA*** in general.

21. How much do you agree or disagree with each of the following statements about ***TEST-RA***? **[ROTATE]**

	Completely disagree	Somewhat disagree	Neither agree nor disagree	Somewhat agree	Completely agree
TEST-RA increases the ability to predict which patients will not respond to anti-TNF therapy					
TEST-RA results in faster time to reach remission or low disease activity state					
TEST-RA increases patient satisfaction					
TEST-RA makes it easier to rule out anti-TNF therapies and to start a patient on another biologic or a JAK inhibitor					
TEST-RA reduces the amount of money spent by patients on ineffective treatments					

22. How helpful is ***TEST-RA*** when deciding whether to start a patient on an anti-TNF therapy?
() Not at all helpful
() Somewhat helpful
() Helpful
() Very helpful

23. How helpful is ***TEST-RA*** when deciding whether to start a patient on other biologics (not anti-TNF therapies) or JAK inhibitors?

() Not at all helpful
() Somewhat helpful
() Helpful
() Very helpful

24. How does ***TEST-RA*** impact your confidence making prescribing decisions for anti-TNF therapies, other biologics, or JAK inhibitors?
    () No change in confidence
    () Somewhat more confident
    () More confident
    () Much more confident

25. What kind of evidence would you want to see before incorporating ***TEST-RA*** into your practice? Please select all that apply.
    [] Scientific journal publications showing clinical utility
    [] Recommendations from thought leaders or peers
    [] FDA approval or recommendation
    [] Recommendation from ACR (American College of Rheumatology) or EULAR (European League Against Rheumatism)
    [] Payer coverage
    [] Other: Please specify ___________

26. Do you believe insurers/payers should provide full coverage for molecular signature tests such as ***TEST-RA***?
    () Yes
    () No

Thank you for your responses. The following questions are for demographic purposes only.

27. What is your gender?
    () Male
    () Female

28. What is your race or ethnicity? Please select all that apply.
    [] White
    [] Black or African American
    [] Hispanic or Latino
    [] American Indian or Alaska Native
    [] Asian
    [] Native Hawaiian or Other Pacific Islander
    [] Other

29. What is your practice setting?
    () Academic
    () Non-Academic

30. What is your practice type?
    () Solo
    () Single specialty
    () Multispecialty

31. Which of the following apply to your practice? Please select all that apply.
    [] My practice is connected with a hospital/hospital system
    [] My practice is part of an IDN (Integrated Delivery Network)
    [] My practice is part of an ACO (Accountable Care Organization)
    [] None of the above

32. What is your practice's geographic location?
    () Rural (fewer than 50,000 people)
    () Suburban (50,000-149,999 people)
    () Urban (150,000 or more people)

Thank you. We appreciate your time and assistance.
